# Effects of a continuous remote care intervention including nutritional ketosis on kidney function and inflammation in adults with type 2 diabetes: a *post-hoc* latent class trajectory analysis

**DOI:** 10.3389/fnut.2025.1609737

**Published:** 2025-06-06

**Authors:** Shaminie J. Athinarayanan, Caroline G. P. Roberts, Stephen D. Phinney, Thomas Weimbs, Allon N. Friedman, Jeff S. Volek

**Affiliations:** ^1^Virta Health, Denver, CO, United States; ^2^School of Medicine, University of California, Davis, Davis, CA, United States; ^3^Department of Molecular, Cellular and Developmental Biology, University of California, Santa Barbara, Santa Barbara, CA, United States; ^4^Department of Medicine, Indiana University School of Medicine, Indianapolis, IN, United States; ^5^Department of Human Sciences, The Ohio State University, Columbus, OH, United States

**Keywords:** nutritional ketosis, type 2 diabetes, eGFR slope, inflammation, dose-response relationship

## Abstract

**Introduction:**

Diabetic nephropathy (DN), a common complication of type 2 diabetes (T2D), is characterized by declining kidney function and an increased risk of end-stage kidney disease (ESKD). Slowing the decline in estimated glomerular filtration rate (eGFR) significantly reduces ESKD risk. While pharmacological treatments, such as SGLT2i, have demonstrated renoprotective effects, emerging evidence suggests that low-grade ketosis may mediate these benefits, and therefore be accessible through lifestyle modification.

**Methods:**

This post-hoc analysis evaluates the impact of a very low-carbohydrate intervention including nutritional ketosis, delivered through a continuous care intervention (CCI), on eGFR slope and inflammation over two years. The analysis included 262 T2D participants in the CCI group and 87 in the usual care (UC) group. The primary aim was to assess the relationship between blood *β*-hydroxybutyrate (BHB) and eGFR slope. A secondary aim explored changes in inflammatory markers including high sensitivity C-reactive protein (hs-CRP) and neutrophil-lymphocyte ratio (NLR). Latent class trajectory modeling was used to categorize ketosis adherence classes in the CCI group based on longitudinal BHB levels.

**Results:**

CCI participants experienced a significant eGFR slope increase of 0.91 mL/min/1.73m^2^/year, compared to a decline in UC (−0.68 mL/min/1.73m^2^/year). Greater mean BHB at 365 days (*β* = 0.1, *p* = 0.002) was independently associated with greater eGFR improvement that persisted after adjusting for demographics, weight change and baseline medication use. A dose–response relationship emerged between ketosis classes and eGFR improvement, particularly among participants with baseline eGFR <90 mL/min/1.73m^2^. Higher ketosis adherence also correlated with significant reductions in inflammatory markers, such as NLR and hsCRP, suggesting anti-inflammatory benefits.

**Conclusion:**

This analysis highlights nutritional ketosis as a potential non-pharmacological approach to improve or stabilize eGFR and reduce inflammation in T2D. Randomized controlled trials are needed to validate these findings and assess the synergistic effects of ketogenic diets combined with pharmacotherapies to optimize kidney outcomes in chronic kidney disease.

## 1 Introduction

Diabetic nephropathy (DN) is a common complication of diabetes and is usually associated with an increased risk of kidney failure, cardiovascular disease and death. A decline in the slope of the estimated glomerular filtration rate (eGFR) portends poorer outcomes (1) and has been designated as a valid surrogate endpoint for kidney failure (2, 3, 4). A slower decline in the eGFR slope decline by 0.75 mL/min/1.73m^2^ is associated with a 21% reduction in end stage kidney disease (ESKD) (2, 3, 4) as demonstrated in a wide variety of studies (2–7).

The antiglycemic medication class of sodium glucose cotransporter 2 inhibitors (SGLT2i) was shown to slow eGFR slope decline and reduced clinically relevant kidney-related outcomes including ESKD in several large, randomized trials (8–10). Because the effect of SGLT2i kidney-related outcomes in these trials was observed to be independent of glycemic control, it was hypothesized that the renoprotective mechanism of SGLT2i may arise from medication-induced ketosis (10, 11, 12). In mechanistic animal studies, it was shown that the renoprotective effect of SGLT2i treatment depends on the production of ketone bodies and that at least part of the effect involves inhibition of mTOR signaling (13). It is possible that this SGLT2i-induced low-grade ketone production elicits renoprotective benefits through its pleiotropic effects as a signaling molecule and as an alternative energy efficient fuel for the kidney that helps restore mitochondrial function (14). Additionally, the anti-inflammatory, antifibrotic and antioxidative effects of the main ketone, beta-hydroxybutyrate (BHB), may also directly benefit the kidney (11, 12, 14, 15).

Although a well-formulated ketogenic diet (WFKD) is a lifestyle intervention option for treating or reversing type 2 diabetes (T2D), it is cautioned against for individuals with underlying impaired kidney function despite the availability of studies showing that a carbohydrate restricted diet is safe or even beneficial in people with moderately diminished kidney function (16). Our non-randomized study of patients with T2D reported improvements in glycemia, body weight and other cardio-metabolic markers including a significant increase in eGFR over two years in those receiving a very low carbohydrate diet targeting nutritional ketosis via a continuous care intervention (CCI) (17). There were similar significant improvements in high sensitivity C-reactive protein (hsCRP) and white blood cell (WBC) counts at 2 years in the CCI arm. Improvements in eGFR, hsCRP and WBC were not observed in the usual care (UC) arm (17). However, it is unknown whether the improvement in eGFR was related to glycemic, weight loss and blood pressure improvements observed in CCI, and whether it is driven by ketone-mediated effects. In this post-hoc analysis, we examined the impact of CCI and diet-induced euketonemia (18) on the kidney function markers, eGFR slope and on inflammation, including the neutrophil-lymphocyte ratio (NLR), an inflammatory marker highly correlated with DN.

## 2 Materials and methods

### 2.1 Study design and participants

The study was an open-label, non-randomized study on participants with type 2 diabetes (T2D) receiving two different treatments, continuous care intervention (CCI) and usual care (UC) (17). The CCI is the treatment arm that consisted of dietary intervention and remote continuous care using telemedicine, while the UC arm received standard of care. The CCI cohort was advised to follow a very low carbohydrate intervention to achieve and sustain nutritional ketosis, i.e., <30 g/day of total carbohydrate in the beginning of the intervention and personalized subsequently based on the individuals’ health goals and carbohydrate tolerance. Protein intake was targeted at a level of 1.5 g/kg of reference body weight, and fat intake targeted to achieve satiety. These participants were monitored telemedically using a web-based software application (app) by their remote care team consisting of a health coach and a healthcare provider. The app was used to upload weight, fingerstick blood glucose and BHB to monitor the patients’ adherence in the diet, their clinical progress, side effects and management of any medication adjustments. BHB values were measured using the Precision Xtra meter (Abbott, Alameda, CA), which has been reported to have a coefficient of variation (CV) of less than 10% across BHB concentrations. BHB values ranging from 0.5 to 5.0 mM are considered within the euketonemia range; however, any value greater than 0.3 mM is still a meaningful indicator that an individual is restricting carbohydrates to a degree that physiologically shifts the body into ketosis (18). The co-primary endpoints of the study were weight and T2D status. Patients were enrolled in the study from August 2015 to May 2016 for a two-year longitudinal assessment. The results from the initial core 2-year study along with the study design, inclusion and exclusion criteria were previously published (17, 19). This post-hoc analysis assesses the intervention effect on 2-year eGFR slope, eGFR stages, and inflammation markers.

### 2.2 Outcomes

There were 262 and 87 participants in the CCI and UC arms, respectively. Kidney-relevant outcomes including serum creatinine, complete blood count (CBC), and hsCRP were collected at baseline, 1 and 2 years. Demographics (age, gender, race, diabetes duration) and other ancillary variables including body mass index (BMI), weight, hemoglobin A1c (HbA1c), systolic blood pressure (SBP), fasting insulin, white blood cell (WBC) and blood urea nitrogen (BUN) that were previously reported in the full CCI and UC cohorts were also included in this post-hoc analysis. eGFR was calculated using the 2021 race-free Chronic Kidney Disease Epidemiology Collaboration (CKD-EPI) creatinine-based equation (20). Neutrophil-lymphocyte ratio (NLR) was calculated by dividing absolute neutrophil count by the absolute lymphocyte count.

### 2.3 Aims

The primary aim of this post-hoc analysis was to assess the relationship between blood BHB and eGFR slope. The secondary aim was to explore changes in inflammatory markers. Other ancillary aims were to assess changes in eGFR and eGFR stage transition from baseline to 2 years among those with baseline eGFR < 60 mL/min/1.73m^2^.

### 2.4 Statistical analysis

Comprehensive details on the statistical analysis, including the latent class trajectory modeling, linear mixed effect model (LMM), and multiple linear regression analyses, are included in the supplementary statistical method section. The guidelines for reporting on latent trajectory studies (GRoLTS) checklist, as recommended by the Equator Network, are included in the supplementary materials (21).

#### 2.4.1 eGFR slope in CCI versus UC

First, we assessed the difference in eGFR total slope from baseline to 2 years between CCI and UC using the linear mixed effect model in all participants and in the subcohort of participants with baseline eGFR <90 mL/min/1.73m^2^.

#### 2.4.2 Association of eGFR improvement with predictors

Given that mean eGFR improvement in CCI was greatest between baseline and 365 days, and remained statistically unchanged from 365 to 730 days, we assessed predictors of eGFR change at 365 days, including mean change in weight, HbA1c, SBP, fasting insulin, HOMA-IR, fasting glucose and lower extremity lean mass at 365 days and mean BHB over 1 year, using multiple linear regression adjusted for demographics and baseline characteristics. As previously published, antiglycemic medications (e.g., SGLT2i, GLP1-RA) (19) were largely deprescribed or unchanged at 1 year, with no significant changes in angiotensin-converting enzyme inhibitors (ACEi) and angiotensin II receptor blockers (ARBs) (22), and no participants on mineralocorticoid receptor antagonists. Sensitivity analysis included baseline use of these medications in the model. Details on the analysis methods are provided in the supplementary methods.

#### 2.4.3 Latent class trajectory modeling (LCTM)

To understand the role of blood BHB on eGFR slope, we explored longitudinal ketone trajectories from baseline to 2 years in the CCI arm using latent class trajectory modeling (LCTM) analysis and assessed eGFR slope in the ketosis classes. Fingerstick BHB logging from baseline to 2 years was used for the LCTM analysis. Instead of using mean BHB data, we used BHB logging data as a count variable (Supplementary material). CCI participants who stayed in the intervention up to 12 weeks and had ketone loggings at least in two of the 8 time periods were included in the LCTM (*n* = 248). We applied the lcmm function from the R lcmm package for the modeling. After selecting the most appropriate link function (see details in the Supplementary Appendix A statistical method; Supplementary Table A1), we then fitted a series of models from 2 through 6 latent classes. Models were estimated using the extended Marquardt algorithm, and missing data were addressed through maximum likelihood estimation. We then assessed and compared the goodness of fit measures, Bayesian information criteria (BIC) and Akaike information criteria (AIC). Then, to select the appropriate model with the optimal number of classes, we assessed the discrimination power of the models using their entropy values and also assessed the relevance of the identified trajectories using their assigned posterior probability.

#### 2.4.4 eGFR slope in ketosis classes within CCI versus UC

We calculated the baseline to 2 years eGFR slopes in the ketosis classes and assessed the difference from UC using the linear mixed effect model among all participants and in a subcohort of participants with baseline eGFR < 90 mL/min/1.73m^2^.

#### 2.4.5 eGFR changes and category transition among those with stage 3 kidney disease

To explore clinically meaningful improvement, we then assessed eGFR changes from baseline to 2 years in a subcohort of CCI participants with stage 3 kidney disease and/or eGFR < 60 mL/min/1.73m^2^ at baseline. We also assessed the proportion of participants that transitioned from stage 3a CKD at baseline to higher (stage 3b or 4) or to lower CKD (stage 2 or less) categories.

#### 2.4.6 Inflammatory markers and blood urea nitrogen

We assessed the changes in the neutrophil-to-lymphocyte ratio (NLR) from baseline to 2 years in both the CCI and UC, across the entire cohort and in a subcohort with abnormal NLR values ≥2.5, using linear mixed effects models. Additionally, we evaluated hsCRP, WBC, NLR (in the entire cohort and a subcohort with baseline abnormal NLR values ≥2.5) and BUN within the CCI ketosis trajectory classes and UC groups, employing the same statistical models.

## 3 Results

Table 1 lists the baseline demographics and variables for the CCI and UC groups, as well as for CCI participants divided by ketosis trajectory classes. In general, no significant differences were observed between CCI ketosis trajectory classes versus UC except for baseline BMI, weight, WBC, percentage of Caucasian and age. CCI sustained nutritional ketosis class had slightly greater baseline BMI and weight compared to UC and other CCI KT classes.

**Table 1:** Demographics and baseline characteristics of the CCI, the different ketosis classes identified within CCI, and the UC group, including the respective mean BHB levels and the percentage of days with BHB ≥ 0.3 mM, are described from baseline to 2 years across the various CCI ketosis classes.

Demographics and baseline characteristics		Whole CCI cohort	Whole UC cohort	CCI trajectory classes				
				Sustained nutritional ketosis	Moderate nutritional ketosis	Low Nutritional ketosis	Unsustained nutritional ketosis	*P*
Baseline N		248	87	17	99	105	27	
One year N		218	78	17	89	93	19	
Two years N		194	68	16	81	80	17	
Mean BHB				consistently 1 mM	around 0.7 mM and dropping to 0.5 mM	around 0.3 to 0.4 mM	around 0.3 mM and dropping to 0.1 mM	
Percentage days of logging BHB ≥ 0.3 mM				consistently >90% of days	around 80–90% and slowly dropping to 70% of days	around 50–60% and slowly dropping to 30% of days	less than 10% of days	
Age, years [mean (SD)]		53.85 (8.36)	52.33 (9.52)	56.29 (8.33)	55.32 (8.08)	52.57 (8.42)	51.85 (8.28)	0.04
Gender (%)	Male	86 (34.7)	36 (41.4)	6 (35.3)	34 (34.3)	34 (32.4)	12 (44.4)	0.63
	Female	162 (65.3)	51 (58.6)	11 (64.7)	65 (65.7)	71 (67.6)	15 (55.6)	
Race and ethnicity (%)	Caucasian	231 (93.1)	87 (100.0)	17 (100.0)	95 (96.0)	96 (91.4)	23 (85.2)	0.01
	Non-Caucasian	17 (6.9)	0 (0.0)	0 (0.0)	4 (4.0)	9 (8.6)	4 (14.8)	
Diabetes duration, years [mean (SD)]		8.34 (7.14)	7.85 (7.32)	7.71 (5.99)	9.51 (6.96)	7.35 (7.43)	8.26 (7.03)	0.29
Baseline BMI, kg/m^2^ [mean (SD)]		40.52 (8.53)	36.72 (7.26)	43.59 (7.46)	40.38 (8.63)	39.87 (7.86)	41.75 (11.11)	0.002
Baseline weight, lbs. [mean (SD)]		258.34 (56.28)	232.87 (48.82)	277.23 (58.62)	257.90 (59.91)	252.72 (51.42)	270.80 (58.54)	0.001
One year weight, lbs. [mean (SD)]		222.78 (48.96)	241.05 (53.99)	224.81 (43.50)	215.87 (51.28)	224.74 (44.38)	248.12 (58.21)	0.001
Two years weight, lbs. [mean (SD)]		225.19 (47.51)	243.51 (55.66)	207.01 (18.48)	222.22 (50.75)	229.77 (45.85)	239.18 (57.75)	0.001
Baseline HbA1c, % [mean (SD)]		7.60 (1.51)	7.64 (1.76)	7.44 (1.78)	7.71 (1.55)	7.53 (1.49)	7.56 (1.28)	0.92
Baseline eGFR, mL/min/1.73m^2^ [mean (SD)]		88.93 (19.02)	88.17 (19.36)	89.82 (16.21)	87.59 (20.26)	89.67 (19.18)	90.41 (15.64)	0.92
Baseline creatinine, mg/dL [mean (SD)]		0.88 (0.24)	0.91 (0.25)	0.86 (0.22)	0.89 (0.26)	0.88 (0.23)	0.89 (0.21)	0.89
Baseline hsCRP, mg/dL [mean (SD)]		8.61 (14.74)	8.89 (8.62)	7.91 (6.37)	9.43 (21.54)	8.12 (7.49)	7.91 (7.63)	0.96
Baseline WBC, k/cumm [mean (SD)]		7.18 (1.83)	8.14 (2.39)	6.87 (1.38)	7.27 (1.79)	7.10 (1.87)	7.40 (2.10)	0.004
Baseline NLR [mean (SD)]		2.32 (0.92)	2.25 (0.91)	2.47 (0.80)	2.33 (0.88)	2.35 (1.00)	2.11 (0.84)	0.66
Baseline BUN, mg/dL [mean (SD)]		16.96 (6.66)	16.05 (6.25)	17.50 (6.10)	17.81 (7.48)	16.30 (5.96)	16.07 (6.37)	0.34
Baseline fasting insulin, mIU/L [mean (SD)]		28.65 (23.82)	29.11 (24.85)	31.57 (23.15)	27.30 (26.25)	30.03 (23.16)	26.13 (17.21)	0.88
Baseline SBP, mmHg [mean (SD)]		132.24 (13.94)	129.80 (13.61)	134.24 (13.49)	132.14 (13.22)	130.90 (14.69)	136.77 (13.45)	0.20
Baseline eGFR stages (%)	1	137 (55.2)	43 (49.4)	11 (64.7)	52 (52.5)	58 (55.2)	16 (59.3)	0.93
	2	88 (35.5)	36 (41.4)	4 (23.5)	38 (38.4)	36 (34.3)	10 (37.0)	
	3a-b	22 (8.9)	8 (9.2)	2 (11.8)	9 (9.1)	10 (9.5)	1 (3.7)	
	4	1 (0.4)	0 (0.0)	0 (0.0)	0 (0.0)	1 (1.0)	0 (0.0)	

The *p*-value listed represents differences between CCI ketosis trajectory classes and the UC as reference category. ANOVA was used to assess differences in continuous outcomes, and the chi-square test was used for categorical outcomes. Abbreviations: CCI, continuous care intervention; UC, usual care; BHB, β-hydroxybutyrate; SD, standard deviation; HbA1c, hemoglobin A1c; eGFR, estimated glomerular filtration rate; BMI, body mass index; WBC, white blood cells; hsCRP, high sensitivity C-reactive protein; NLR, neutrophil-lymphocyte ratio; BUN, blood urea nitrogen; SBP, systolic blood pressure.

### 3.1 eGFR slope in CCI versus UC

CCI was associated with a significant increase in eGFR from baseline to 2 years with an eGFR slope 0.91 mL/min/1.73m^2^/year (*p* = 0.03). In contrast, UC was associated with a non-significant decrease in eGFR (−0.68 mL/min/1.73m^2^/year). The between group difference was 1.59 (−3.23, 0.05) mL/min/1.73m^2^/year and of borderline statistical significance (*p* = 0.06). Similarly, in a subcohort of participants with baseline eGFR<90 mL/min/1.73m^2^, the difference in the annual rate of change between CCI vs. UC in eGFR was 1.98 (−4.74, 0.79) mL/min/1.73m^2^/year, where CCI had a positive eGFR slope of 3.04 mL/min/1.73m^2^/year (*p* < 0.001).

### 3.2 Association of eGFR improvement with predictors

The results of the multiple regression analyses are presented in Table 2, focusing on predictors previously reported in the literature. Other potential predictors, such as change in lower extremity lean mass, HOMA-IR, and fasting glucose at 365 days, were not significantly associated with change in eGFR at 365 days. After adjusting for baseline predictors (which remained consistent across all models), only mean change in weight (Model 1) and mean BHB (Model 5) at 365 days were significantly associated with mean change in eGFR at 365 days in the models where change-related predictors were included individually. In Model 6, which included both mean delta weight and mean BHB over 1 year, only mean BHB over 1 year (*β* = 0.1, *p* = 0.009) was associated with mean change in eGFR at 365 days, with greater mean BHB positively associated with greater mean change in eGFR at 365 days. Additionally, higher baseline eGFR (*β* = 0.8, *p* < 0.001) and shorter diabetes duration (*β* = −0.1, *p* = 0.01) were associated with a greater mean change in eGFR at 365 days.

**Table 2:** Multiple linear regression models for association of different baseline and change in independent factors with change in eGFR at 365 days.

Parameters	Model 1		Model 2		Model 3		Model 4		Model 5		Model 6		Final Model	
Baseline Predictors	*β*	*p*-value	*β*	*p*-value	*β*	*p*-value	*β*	*p*-value	*β*	*p*-value	*β*	*p*-value	*β*	*p*-value
Age	−0.07	0.2	−0.03	0.49	−0.04	0.46	−0.04	0.48	−0.04	0.38	−0.06	0.21	−0.09	0.07
Gender	−0.04	0.36	−0.05	0.25	−0.02	0.7	−0.02	0.68	−0.01	0.76	−0.03	0.49	−0.01	0.88
Race	0.00	0.96	−0.01	0.9	−0.03	0.58	−0.01	0.92	−0.01	0.85	0.01	0.77	−0.01	0.89
Diabetes duration	−0.10	0.02	−0.12	0.01	−0.12	0.01	−0.10	0.03	−0.12	0.01	−0.11	0.01	−0.10	0.02
Baseline eGFR	0.77	<0.001	0.78	<0.001	0.77	<0.001	0.77	<0.001	0.77	<0.001	0.78	<0.001	0.77	<0.001
BMI			0.05	0.23	0.02	0.71	0.04	0.42	0.02	0.61				
Baseline weight	−0.13	0.27									−0.03	0.56	−0.02	0.64
Baseline HbA1c			−0.08	0.3										
Baseline systolic blood pressure					0.02	0.8								
Baseline fasting insulin							−0.01	0.83						
Baseline SGLT2i use													0.07	0.10
Baseline GLP1-RA use													−0.09	0.04
Baseline ARB and ACEi use													0.14	0.002
Change Predictors														
Delta weight	−0.06	0.01									−0.08	0.17	−0.06	0.26
Delta HbA1c			−1.88	0.06										
Delta systolic blood pressure					0.04	0.53								
Delta fasting insulin							0.02	0.78						
Mean ketone 1 to 365 days									0.12	0.01	0.12	0.01	0.12	0.002

As previously published, antiglycemic medications (e.g., SGLT2i, GLP1-RA) (19) were largely deprescribed or remained unchanged at 1 year, with no significant changes in the use of angiotensin-converting enzyme inhibitors (ACEi) and angiotensin II receptor blockers (ARBs) (22), and no participants were on mineralocorticoid receptor antagonists. In the cohort used for this regression analysis, 10.9% of participants were on SGLT2i and 13.7% were on GLP-1 RAs at baseline. GLP-1 RA use remained unchanged at 1 year, with 13.3% of participants continuing therapy. In contrast, SGLT2 inhibitors were deprescribed in most participants as part of the individualized medication management protocol in the CCI group, with only 0.9% remaining on SGLT2i at 1 year. In the final multivariate sensitivity model, mean BHB at 365 days (*β* = 0.1, *p* = 0.002) remained significantly associated with change in eGFR at 365 days, even after adjusting for baseline medication use of SGLT2i, GLP1-RA, and ARBs/ACEi (Table 2). Both baseline use of GLP1-RA and ARBs/ACEi were associated with mean change in eGFR at 365 days, with ARBs/ACEi use positively associated and GLP1-RA use negatively associated with a greater mean change in eGFR at 365 days.

### 3.3 The CCI group can be sub-classified into four ketosis classes based on achieved BHB levels

**Figure 1:**
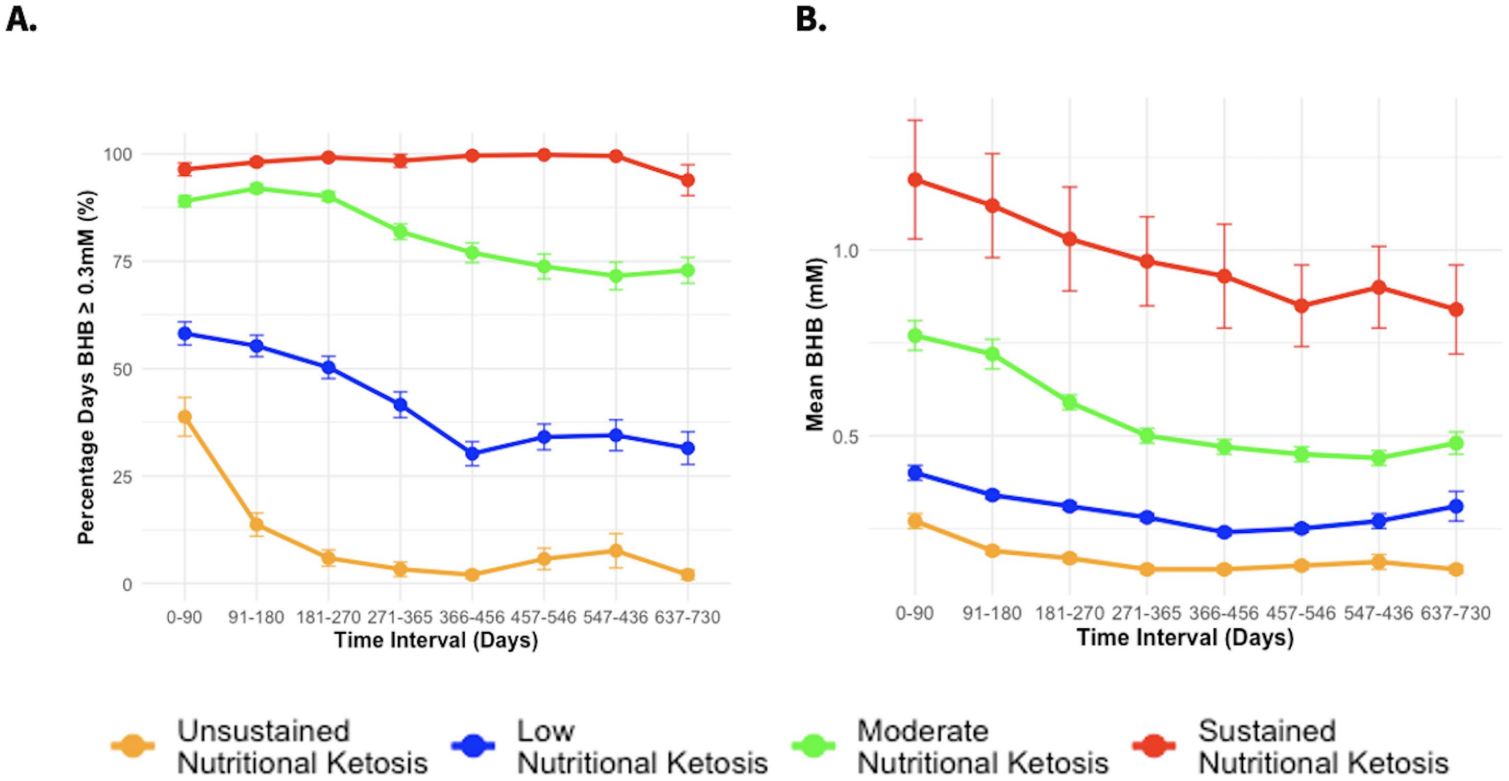
**(A)** Percentage days of logging BHB ≥ 0.3 mM from baseline to 2 years in different CCI ketosis classes. **(B)** Mean BHB from baseline to 2 years in different CCI ketosis classes.

To further investigate the possible association between BHB levels and reno-protection, we subclassified the CCI group based on achieved BHB levels. Details on model specification, parameter and selection are included in the Supplementary Appendix A1 Statistical Results and Supplementary Table A1. According to the model specification, the model with 4 latent ketosis classes was deemed to be the best fit (i.e., lower BIC, AIC, and SABIC values) when compared to models with 3 or lower and 5 or more latent classes (Supplementary Tables A2, A3). The entropy of the model was 0.74 indicating good separation between the classes. Supplementary Table A4 includes a completed GRoLTS (Guidelines for Reporting on Latent Trajectory Studies) checklist for the latent class trajectory analysis. The ketosis classes were identified as unsustained nutritional ketosis (UNK, *n* = 27), low nutritional ketosis (LNK, *n* = 105), moderate nutritional ketosis (MNK, *n* = 99) and sustained nutritional ketosis (SNK, *n* = 17) as illustrated in Figures 1A,B. Table 1 provides details of the mean BHB and percentage of days logging BHB ≥ 0.3 mM in each of the ketosis trajectory classes.

### 3.4 Dose-dependent effect of ketosis adherence on eGFR slope improvement

**Figure 2:**
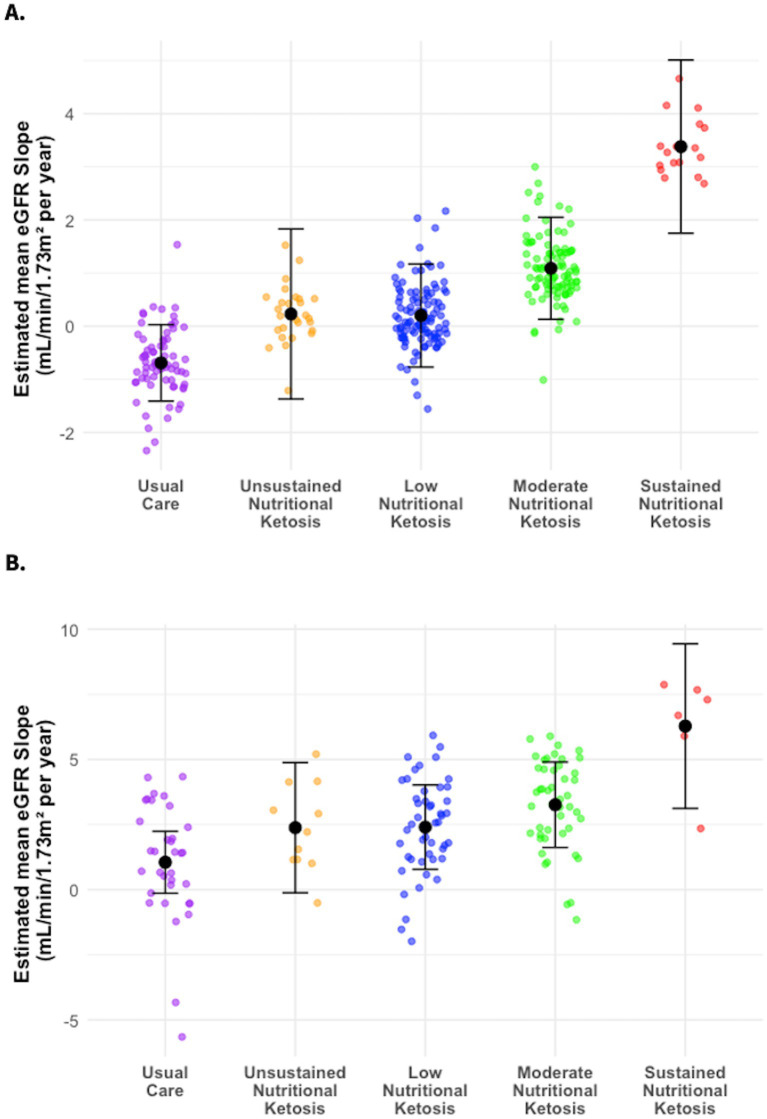
Estimated mean eGFR Slope (mL/min/1.73m^2^ per year) from the linear mixed model by different CCI ketosis classes and usual care (UC). The plot shows the estimated mean eGFR slope with standard error (SE) for each class and UC. **(A)** Whole cohort CCI ketosis trajectory classes and UC. **(B)** Sub cohort CCI ketosis trajectory classes and UC with baseline eGFR <90.

Table 3 presents the estimated eGFR slopes in the different ketosis trajectory classes in comparison to UC. Individuals in the two higher ketosis adherence categories, namely SNK and MNK, had a greater increase in their eGFR slope from baseline to two years relative to those categorized as lower ketosis adherence (LNK and UNK) (Figure 2). Specifically, the eGFR slope within the SNK group was significantly higher compared to the UC group, with an observed between-group difference of 4.06 mL/min/1.73m^2^ annually. Furthermore, analysis revealed a dose–response relationship between the intensity and duration of ketosis, as classified into distinct ketosis classes, and the improvement of eGFR slope (Figure 2A and Table 3). This dose-dependent effect of ketosis adherence on eGFR slope improvement was more pronounced among participants who started off with a baseline eGFR < 90 (Figure 2B and Table 3).

**Table 3:** Two-year eGFR slopes in CCI participants divided into four ketosis classes in the whole CCI cohort, CCI subcohort with baseline eGFR<90 and in UC (control) used as a reference category in the linear mixed effect model.

	eGFR slope (mL/min/1.73m^2^/year)		
Whole cohort CCI divided into 4 ketosis trajectory classes	Mean + SE	Slope difference	*p*-value
Time * Group interaction			0.11
Sustained nutritional ketosis (SNK, *N* = 17)	3.38 ± 2.35	4.06 ± 1.63	0.01
Moderately declining nutritional ketosis (MNK, *N* = 99)	1.09 ± 1.68	1.78 ± 0.96	0.07
Low nutritional ketosis (LNK, *N* = 105)	0.20 ± 1.69	0.88 ± 0.97	0.36
Unsustained nutritional ketosis (UNK, *N* = 27)	0.22 ± 2.32	0.91 ± 1.60	0.57
UC (control)	−0.69 ± 0.72		
Sub cohort CCI with baseline eGFR <90 divided into 4 ketosis trajectory classes			
Time * Group interaction			0.47
SNK (*N* = 8)	6.28 ± 4.35	5.22 ± 3.16	0.10
MNK (*N* = 47)	3.26 ± 2.83	2.21 ± 1.64	0.18
LNK (*N* = 47)	2.40 ± 2.81	1.34 ± 1.62	0.41
UNK (*N* = 11)	2.38 ± 3.69	1.33 ± 2.50	0.60
UC (control)	1.05 ± 1.19		

eGFR slope estimates were obtained from linear mixed-effects models controlling for baseline age, sex, race, insulin use, and diabetes duration. The maximum-likelihood based approach uses all available data, resulting in an intent-to-treat analysis.

### 3.5 eGFR changes and category transition among those with stage 3a or higher kidney disease (eGFR<60)

Among participants with baseline eGFR indicating advanced kidney disease (stage 3a or higher), 23 individuals in the CCI group showed significant improvement, with mean eGFR increasing from 49.9 to 64.7 mL/min/1.73m^2^ at 2 years (*p* < 0.001). Of those with 2-year eGFR data (*n* = 15), 93% were in stage 3 and 7% in stage 4 at baseline. Notably, none progressed to more severe CKD, and 53% improved to stage 2, 7% improved from stage 4 to stage 3, while 40% remained in stage 3.

In contrast, among 8 individuals with mild to moderate kidney disease in the UC group, 6 had 2-year eGFR data. Only 17% improved to stage 2, 67% remained in stage 3, and 17% worsened to stage 4.

### 3.6 Inflammatory markers and blood urea nitrogen

At 2 years, the CCI cohort experienced significant reductions in hsCRP, WBC, and NLR levels, while no significant changes were observed in the UC group (Table 4). Reductions in inflammation markers were observed across all four CCI ketosis trajectory classes, with a clear dose–response relationship. Participants with higher ketosis adherence (SNK and MNK) showed the greatest improvements in hsCRP (−57.5% and −32.0%, respectively) and WBC (−20.6% and −10.7%, respectively) compared to those with lower adherence (LNK: hsCRP -40.7%, WBC -2.9%; UNK: hsCRP -28.0%, WBC -3.7%) (Table 4). Similarly, NLR decreased significantly in the CCI cohort (−8.5%) and in a subcohort with baseline NLR ≥ 2.5 (−15.2%), with no significant changes in UC. Improvements in NLR also followed a dose–response pattern, with greater reductions in higher ketosis adherence categories. These results suggest a robust, dose-dependent anti-inflammatory effect of nutritional ketosis. BUN levels remained stable and within the normal range across all CCI classes and UC (Table 4).

**Table 4:** Inflammatory markers by CCI ketosis trajectory classes versus UC from baseline to 2 years.

Variables	Timepoints	Reference	CCI ketosis trajectory classes			
		UC	Unsustained nutritional ketosis	Low nutritional ketosis	Moderate nutritional ketosis	Sustained nutritional ketosis
		Mean ± SE				
hs C-reactive protein (mg/dL)	Baseline	8.9 ± 0.8	7.3 ± 1.3	7.5 ± 0.7	7.6 ± 0.7	7.3 ± 1.6
	12 months	9.1 ± 0.9	5.3 ± 1.5	5.1 ± 0.7***	4.9 ± 0.7***	5.6 ± 1.6
	24 months	8.3 ± 0.8	5.3 ± 1.4	4.4 ± 0.6***	5.2 ± 0.6***	3.1 ± 1.4***
White blood cell (k/cumm)	Baseline	8.3 ± 0.2	7.2 ± 0.4	7.1 ± 0.2	7.3 ± 0.2	6.8 ± 0.5
	12 months	8.3 ± 0.2	6.8 ± 0.4	6.7 ± 0.2*	6.3 ± 0.2***	5.5 ± 0.5***
	24 months	8.0 ± 0.3	7.0 ± 0.5	6.9 ± 0.2	6.5 ± 0.2***	5.4 ± 0.5***
Neutrophil to lymphocyte ratio (NLR); whole cohort	Baseline	2.3 ± 0.1	2.1 ± 0.2	2.4 ± 0.1	2.3 ± 0.1	2.3 ± 0.2
	12 months	2.4 ± 0.1	2.1 ± 0.2	2.2 ± 0.1	2.1 ± 0.1 *	2.3 ± 0.2
	24 months	2.2 ± 0.1	1.8 ± 0.2	2.2 ± 0.1	2.1 ± 0.1 *	2.0 ± 0.2 *
Neutrophil to lymphocyte ratio (NLR); subcohort >2.5	Baseline	2.9 ± 0.1	2.7 ± 0.2	2.9 ± 0.1	2.8 ± 0.1	2.9 ± 0.2
	12 months	2.9 ± 0.2	2.7 ± 0.3	2.7 ± 0.1 *	2.4 ± 0.1 **	2.7 ± 0.3
	24 months	2.6 ± 0.2	2.1 ± 0.3 *	2.6 ± 0.1 **	2.3 ± 0.1 ***	2.2 ± 0.2 ***
Blood urea nitrogen (BUN) (mg/dL)	Baseline	16.1 ± 0.7	16.1 ± 1.2	16.7 ± 0.6	17.5 ± 0.6	17.1 ± 1.5
	12 months	16.3 ± 0.9	18.0 ± 1.7	19.5 ± 0.8	19.1 ± 0.8	17.9 ± 1.8
	24 months	16.8 ± 0.8	16.6 ± 1.6	18.2 ± 0.7	17.7 ± 0.7	17.4 ± 1.6

Estimated means and standard errors were derived from linear mixed effects models using the maximum-likelihood approach adjusted for within-subject correlation of measures. The following covariates were also included in the models: baseline age, sex. Race, body mass index, baseline diabetes medication use, and diabetes duration.

***p-value <0.001; **p-value <0.01; *p-value <0.05.

## 4 Discussion

### 4.1 eGFR slope

In this study, we demonstrated for the first time that a very low carbohydrate intervention leading to nutritional ketosis is associated with an increase in the rate of eGFR change of 0.91 mL/min/1.73m^2^/year from baseline to two years in participants with type 2 diabetes. This exceeded the recognized threshold of a clinically meaningful eGFR slope increase of 0.75 mL/min/1.73m^2^ (3, 4). Of note, the increase in the eGFR slope was more evident at higher levels of ketosis and at progressively lower baseline eGFR levels (baseline eGFR <90 mL/min/1.73m^2^). These findings raise the possibility that a very low carbohydrate intervention could be used to stall or even reverse the progression of diabetic nephropathy to end-stage kidney disease (3, 4).

No other nutritional intervention has demonstrated a similarly positive impact on the eGFR slope. The CORDIOPREV study reported an annual eGFR decline of approximately −1 mL/min/1.73m^2^ with a Mediterranean diet over five years (23), while the MDRD study observed declines of −2.1 and −3.2 mL/min/1.73m^2^ per year in patients with moderate to severe kidney dysfunction following low-protein and very low-protein diets, respectively (24). Similarly, a post-hoc analysis of the Look AHEAD trial found declines of −0.86 and −0.93 mL/min/1.73m^2^ per year over 10 years in the intensive lifestyle intervention (ILI) and diabetes support and education (DSE) groups (25). These studies, however, benefited from longer follow-up periods compared to ours and differed in terms of the study population. Pharmaceutical trials with SGLT2i (8–10), GLP1-RA (26, 27), and finerenone (28) in participants with pre-existing CKD at high risk for progression also reported negative eGFR slopes in both treatment and placebo groups. However, the difference in eGFR decline between the groups ranged from 0.8 to 2.0 mL/min/1.73m^2^/year, with the placebo arms experiencing greater declines, and the drugs effectively mitigating the rate of decline. In contrast, our nutritional intervention, which targeted nutritional ketosis, uniquely demonstrated a positive eGFR slope of 0.91 mL/min/1.73m^2^/year in patients with relatively intact kidney function. This improvement was particularly notable in participants with a baseline eGFR <90 mL/min/1.73m^2^, where the difference between the intervention and usual care groups was 1.97 mL/min/1.73m^2^/year. Moreover, the absence of significant changes in blood urea nitrogen suggests that the positive eGFR slope was not attributable to hyperfiltration from increased protein intake, highlighting the unique renoprotective potential of this approach.

Healthy individuals in the general population usually have an eGFR slope of approximately −1 mL/min/1.73m^2^/year (29), while patients with T2DM generally have a more rapid decline (30). Our intervention appears to have staved off and even reversed such a decline in the eGFR slope especially among those with lower eGFRs. This trend was consistent among those who started with an eGFR <60 mL/min/1.73m^2^ at baseline, where these patients not only experienced an increase in their eGFR but 53% of them reversed their eGFR staging from stage 3 to stage 2. Nutritional interventions that have the potential to slow the eGFR decline or maintain the eGFR decline to a rate similar to the general population are associated with improved kidney outcomes. Two different post-hoc analyses of the Look AHEAD study data revealed significant benefits for kidney health (31, 32). An earlier study demonstrated that participants in the ILI group had a reduced risk (HR 0.69, 95% CI; 0.55 to 0.87; difference of 0.27 cases per 100 person-years) of developing high-risk CKD, as defined by the 2013 KDIGO classification, compared to the DSE arm (31). A more recent study reported a significant reduction in eGFR < 45 mL/min/1.73m^2^ in the ILI arm compared to the DSE arm during the active intervention phase, with a hazard ratio of 0.80 (95% CI; 0.66 to 0.98) (32).

None of these previous nutritional or pharmacological intervention studies achieved reversals of the eGFR slopes to positive values. Even the best therapies only slow the rate of eGFR decline. The main difference between our nutritional intervention and previous nutritional interventions is that our very low-carbohydrate approach leads to sustained nutritional ketosis in many of the participants. This suggests that the metabolic state of ketosis, characterized by physiologic levels of ketones, may be the key to the observed reno-protective effects. Ketones, particularly BHB, are believed to exert reno-protective benefits through multiple mechanisms, including serving as an efficient alternative energy source for the kidney, reducing oxidative stress, and exhibiting anti-inflammatory and antifibrotic effects, which collectively help preserve kidney function and mitigate damage (33). However, it’s important to consider whether similar benefits would be seen with exogenous BHB supplementation or if sustained endogenous ketosis reflects broader metabolic improvements, particularly reduced hyperinsulinemia, which is known to drive inflammatory pathways (e.g., NLRP3 via PI3K) and other renal stressors (34, 35). Endogenous ketosis likely signals reduced insulin exposure over time, which in turn impacts markers like vitamin D metabolism (36), and inflammatory cytokines (35). Additionally, carbohydrate restriction—which induces endogenous ketosis—may directly ameliorate hyperglycemia and reduce the formation of advanced glycated end products implicated in kidney injury (37). Consequently, the combined effects of carbohydrate restriction, elevated endogenous BHB levels, and lower insulin may contribute to improved eGFR, although further research is required to delineate the independent role of BHB.

### 4.2 Association of ketones with eGFR improvement

The improvement in eGFR in the CCI group was most notable between baseline and one year, coinciding with the period of highest adherence to the carbohydrate restriction. Similar to the observed eGFR improvement trend, a greater increase in mean BHB levels from baseline to 365 days was significantly associated with eGFR improvement, along with shorter diabetes duration, higher baseline eGFR and baseline use of GLP1-RA and ACEi/ARBs after accounting for body weight changes at one year. While GLP-1 RA use at baseline was independently associated with changes in eGFR at 1 year, no such association was observed for SGLT2i, likely due to the limited sample size, substantial deprescription of SGLT2i during the study, and the resulting variability in exposure. It is also important to note that participants on GLP-1 RAs were primarily using older-generation agents, and thus, this observed association should be interpreted cautiously. Future studies with larger cohorts, consistent medication exposure, and newer GLP-1 RA formulations are needed to validate these findings and explore potential additive effects with nutritional interventions. Likewise, our findings were consistent with two other studies that found kidney function improvement from a low carbohydrate nutritional intervention was not correlated with the degree of weight loss (38, 39). Additionally, one of these studies reported an association between eGFR improvement and a decrease in fasting insulin and systolic blood pressure (39), but a similar association was not observed in our study. However, neither of these studies assessed whether changes in mean ketone levels were associated with eGFR improvement (38, 39) or accounted for mean ketones in the model that included fasting insulin (39). It is possible that the association between the decrease in fasting insulin and eGFR improvement was due to carbohydrate restriction, which helps increase insulin sensitivity and reduce the need for greater insulin secretion (40). Moreover, another recent study reported that ketonuria-an indicator of high level of blood ketone levels - was the only significant factor associated with six-month eGFR improvement in those treated with SGLT2i in both univariate and multivariate linear regression (41).

Like the association analysis, we also found a clear dose–response relationship between ketosis classes and eGFR slope. Individuals with closer dietary adherence and maintenance of ketosis categories had a greater increase in their eGFR slope from baseline to two years compared to those demonstrating lower ketosis adherence groups. This relationship was more evident in those with mild baseline kidney dysfunction. These findings align with the reno-protective effect of SGLT2i, which is associated with the presence of ketonuria (41), and mechanistically depends on the production of BHB (13). The improvement in eGFR was approximately seven-fold greater in those on SGLT2i with ketonuria versus those on SGLT2i without ketonuria (41). Likewise, in genetically driven autosomal polycystic kidney disease (ADPKD) a ketogenic diet resulted in increased eGFR in a randomized-controlled trial (42). A post-hoc analysis of the FDA-accepted outcome measure of height-adjusted total kidney volume (htTKV) showed that patients in the ketogenic diet arm who reached a greater ketosis threshold experienced a greater reduction in htTKV (43). The apparent dose-dependent beneficial relationship between BHB levels and reno-protection suggests that nutritional interventions leading to a deeper level of ketosis may be more effective in DN. Alternatively, or in addition, supplementation with exogenous BHB may be of added benefit which is currently being explored for ADPKD (44).

While the mechanistic and biochemical actions of ketones on kidney function could be attributed to their putative anti-inflammatory, anti-fibrotic, and anti-oxidative effects, it is still unknown whether these effects lead to a reduction in adverse kidney outcomes or events. Several clinical and real-world observational studies have reported improvements in kidney function markers among those following a low or very low carbohydrate diet (38, 39, 45–47), including studies that showed improvements in urine albumin creatinine ratio (UACR) (38) and cystatin C levels (47). Even lowering carbohydrate intake to non-ketogenic levels appears beneficial. Thus far, there is only one study that has reported a significant reduction in the risk of doubling of serum creatinine and development of dialysis dependency, or all-cause mortality in a carbohydrate restricted cohort compared to a standard protein restriction diet (48). One large observational study that assessed the association between carbohydrate intake and mortality in those with CKD reported a reduction in mortality in groups classified as having lower carbohydrate intake (HR of 0.76, 95% CI; 0.62 to 0.93) (49).

### 4.3 Inflammation

The close interrelationship between inflammation and CKD is also well established, with numerous studies highlighting the role of various inflammatory markers and the NLRP3 inflammasome in the progression of kidney damage (50, 51). One such marker, the neutrophil-lymphocyte ratio (NLR), has garnered attention for its predictive value in DN (52, 53). Elevated NLR levels indicate an inflammatory state and are associated with worse renal outcomes in patients with T2D, and with increased UACR (54, 55) and lower eGFR (56) in patients with DN. In our study, we observed a significant reduction in NLR, along with hsCRP and WBC, from baseline to two years that followed a dose–response trend related to adherence and maintenance of ketosis level. Despite NLR being a predictive marker for DN and its progression, there are still no studies directly evaluating the impact of NLR reduction on kidney function improvement or the reduction of adverse kidney outcomes. However, several studies indicate that NLR above certain cutoffs, ranging from 2.2 to 3.0, are most likely associated with diabetic nephropathy (56, 57–59). In the subgroup analysis of those starting with NLR ≥ 2.5, there was a 15% average reduction in NLR, with the average falling below the cutoff after two years. Furthermore, both high ketosis adherence classes experienced the greatest decline in NLR, normalizing the NLR levels to <2.5 at two years.

### 4.4 Strengths and limitations

Our study has several strengths, including the inclusion of two distinct groups that were closely followed for at least two years, allowing us to assess differences in eGFR slope between them. Additionally, ketone levels were routinely monitored in the CCI group using blood ketone meters, with values uploaded through the app. This enabled us to examine the association between ketone exposure and improvements in eGFR. However, the study also has several limitations. It represents a *post hoc* secondary analysis of kidney function markers and was not specifically powered to assess kidney outcomes; thus, the findings should be considered hypothesis-generating. Another key limitation is the lack of detailed information on the underlying causes of CKD and the use of immunosuppressive agents, as the trial was not originally designed to evaluate kidney-specific endpoints. Furthermore, the interpretation of our findings is limited to eGFR improvement; the impact of the intervention on urinary albumin-to-creatinine ratio (UACR) remains unknown. Although urine microalbumin was measured, no significant changes were observed from baseline to two years. The findings from this analysis are limited to patients with type 2 diabetes (T2D). Furthermore, eGFR was estimated using serum creatinine, which can be influenced by weight loss, particularly by changes in lean mass. Rather than using cystatin C or direct measurement via 24-h urinary creatinine clearance. Finally, the moderate sample size may limit the generalizability of the findings.

## 5 Conclusion

In conclusion, our study provides preliminary evidence suggesting a relationship between generation of a ketotic state from a carbohydrate-restricted intervention and reduction in circulating markers of inflammation and stabilization or improvement of eGFR and risk of DN in individuals with T2D. The exploratory data help establish a scientific rationale for a randomized controlled trials to test the effects of a very low carbohydrate diet versus the standard of care on kidney related outcomes in individuals with CKD and on state-of-the art therapy. Furthermore, future research should investigate the additive effects between drugs approved for CKD, such as SGLT2i or GLP-1 receptor agonists, and dietary carbohydrate restriction. Understanding how these medications interact with a very low carbohydrate diet could provide valuable insights into optimizing treatment strategies for CKD patients and potentially enhance the therapeutic benefits.

## Data Availability

The data analyzed in this study is subject to the following licenses/restrictions: data is available upon appropriate request to the corresponding author, accompanied by a detailed proposal outlining how the data will be used. Requests to access these datasets should be directed to Shaminie J. Athinarayanan, shaminie@virtahealth.com.
