# Supplementary material for: Effects of a continuous remote care intervention including nutritional ketosis on kidney function and inflammation in adults with type 2 diabetes: a post-hoc latent class trajectory analysis
Source: Front Nutr. 2025 Jun 6;12:1609737. doi: 10.3389/fnut.2025.1609737 (PMC12178890; doi:10.3389/fnut.2025.1609737)
Supplement: Supplementary file 1 [file Table_1.DOCX]

**Supplementary Statistical Method**

**Linear Mixed Effect Model (LMM)**

**eGFR slope**

In this study, a linear mixed-effects modeling (LMM) was used to quantify the total slope of the eGFR and to discern differential slopes among distinct groups. The analysis assessed the longitudinal change of the eGFR across various groups over the study duration (baseline to 2 years). The models incorporated fixed effects for time, group, and the interaction between time and group- allowing to directly estimate the eGFR slope over time in each group. Four different LMMs were performed including LMM assessing the eGFR slope difference between CCI vs UC, the eGFR slope difference between CCI vs UC in a subset of cohort with a baseline eGFR <90 mL/min/1.73m^2^, the eGFR slope differences between the different CCI ketosis classes vs UC and the eGFR slope differences between the different CCI ketosis classes vs UC in a subset of cohort with a baseline eGFR <90 mL/min/1.73m^2^ . To account for intra-individual variability and the inherent correlation of repeated measurements, random effects were incorporated for both the intercept and time-dependent slope, thus accommodating unique baseline attributes and temporal dynamics for each subject. An unstructured covariance matrix was specified for the random effects to allow for the estimation of unique variance components for each effect and its covariance. Furthermore, relevant covariates including age, gender, race, diabetes duration, insulin use were included in the model to adjust for potential confounding factors.

**Longitudinal changes of hsCRP, WBC, and NLR**

For our analysis, LMM were utilized to (1) explore changes in hsCRP, WBC, and NLR within each CCI ketosis class and UC from the baseline up to the two-year follow-up and (2) to assess differences in outcomes between the groups (CCI ketosis classes vs UC) at the two-year endpoint. These models included fixed effects for time, group allocation (CCI ketosis classes versus UC), and the interaction between time and group, facilitating within-group changes and between-group differences. The models were further refined by incorporating covariates such as age, sex, racial background (with a specific focus on African American versus other racial groups), body Mass Index (BMI), and insulin usage at baseline, to control for their potential confounding effects. Utilizing a maximum likelihood estimation method enabled the inclusion of all available repeated measures data, embodying an intent-to-treat analysis approach. To adequately capture the correlation patterns among the repeated measures for each participant, an unstructured covariance matrix was implemented in all model specifications, ensuring a thorough and precise estimation of the temporal associations.

**Multiple Linear Regression**

Multiple regression analysis was conducted to evaluate the impact of various independent predictors on eGFR changes at 365 days. “The independent factors, change in weight, HbA1c, SBP, fasting insulin and mean BHB at 365 days included in the analysis were selected based on previous empirical evidence or theoretical relevance suggesting potential associations with eGFR changes.” First, independent predictors were included in separate multiple regression models (models 1 to 5), adjusted by gender, age, baseline BMI, baseline eGFR, diabetes duration and baseline predictors as covariates. Any independent predictors that were significant were then included in the final model (model 6), adjusted by gender, age, baseline BMI, baseline eGFR, diabetes duration and baseline predictors as covariates. As previously published, antiglycemic medications such as SGLT2i and GLP-1 receptor agonists (GLP1-RA) were either significantly deprescribed or remained unchanged at 1 year (19). Similarly, no significant changes were reported in the percentage of participants taking medications like angiotensin-converting enzyme inhibitors (ACEi) and angiotensin II receptor blockers (ARBs) at 1 year and none of the participants were taking any mineralocorticoid receptor antagonists (22). A sensitivity analysis was repeated for the final multiple linear regression model, where baseline use of SGLT2i, GLP-1RA, ACEi, and ARBs was included in the model. The results of the multiple regression analysis are reported as beta coefficients with p-values.

**Latent Class Trajectory Analysis (LCTM)**

Latent class trajectory analysis (LCTM) was performed to identify different ketosis trajectory classes in the CCI arm. Days with no BHB logs (null) were considered as zero value. The BHB logging data was used as a count variable. We first assessed the distribution of the total days of logging BHB value ≥ 0.5mM, total days of logging BHB value ≥ 0.3mM, percentage days of logging BHB value ≥ 0.5mM and percentage days of logging BHB value ≥ 0.3mM in these patients. The logging data were stratified into 8 time periods of 3 months interval (0-90, 91-180, 181-270, 271-365, 366-456, 457-546, 547-636, 637-730 days). Three of the logging variables had negative binomial distribution while the percentage days of logging BHB value ≥ 0.3mM showed a left skewed Poisson distribution where greater proportion of the individuals who were logging BHB were logging BHB values ≥ 0.3mM. The total days of logging BHB value ≥ 0.5mM and percentage days of logging BHB value ≥ 0.5mM both had a greater proportion of zero values with a zero-inflated distribution. To avoid the use of variables with zero-inflated distribution, we then selected the variable percentage days of logging BHB value ≥ 0.3mM for the LCTM analysis.

Different trajectories of percentage days of logging BHB value ≥ 0.3mM were identified using the latent class mixed model (lcmm) with the lcmm R package version 4.1.0. Since the variable percentage days of logging BHB value ≥ 0.3mM was non-normally distributed with a Poisson distribution, we applied the lcmm function from the R package for the modeling. The lcmm function uses a modified Marquadt iterative algorithm and maximum likelihood estimation in the model. The percentage days of logging BHB value ≥ 0.3mM was modeled longitudinally as a function of treatment time. An unconditional LCMM was performed without any covariates or predictors to generate raw latent classes for the percentage days of logging BHB value ≥ 0.3mM. First, the latent process mixed model for a 1-class model was fitted with different link functions (linear, beta cumulative function, and quadratic I-splines either with different number of nodes and/or equidistant nodes). To select the appropriate link function, the goodness-of-fit measures AIC and BIC from all these 1-class models were compared (Supplementary Table 1). The model with the lowest AIC and BIC is preferred and this selected link function was used for the subsequent models with increasing numbers of classes.

Based on the 1-class model assessment, we selected the quadratic I-splines function with 7 nodes. We then used this link function to assess models up to 6 classes using parameter estimation generated from the 1-class model, an unstructured variance-covariance matrix of the random effects and a common variance-covariance structure across all latent classes (Supplementary Table 2). A default 100 iterations was used for the Marquadt iterative algorithm, and 100 different points were used to plot the estimated link function. The models (classes =1 to 6) were also refitted using an alternative latent-class specific variance-covariance structure (Supplementary Table 3). We also performed the model under a grid-search function with a maximum of 30 iterations from 100 random vectors of the initial estimation values of a 1-class model. We then assessed and compared the goodness of fit measures, Bayesian information criteria (BIC) and Akaike information criteria (AIC).

**Supplementary Statistical Results**

**Latent Class Trajectory Analysis (LCTM)**

Based on the 1-class model specification, the model with the 7-quant-splines function was considered the best-fitting model for ketone data and was selected to estimate models with an increasing number of classes, up to six classes. Details on model parameters and selection are included in Supplementary Table 1. The model with the 7-quant-splines function had an AIC of -1.97E+03 and a BIC of -1.93E+03, which were the lowest AIC and BIC values compared to the models with alternative link functions. The parameter estimates from the model with the 7-quant-splines function were incorporated into the models with an increasing number of classes. These increasing-number-of-classes models were fitted using two different variance-covariance structures. The results generated from the common variance-covariance structure are listed in Supplementary Table 2, and the results from the alternative class-specific variance-covariance structure are listed in Supplementary Table 3.

Based on the model fit, AIC, and BIC values, the models with the alternative class-specific variance-covariance structure were considered to have a better fit, and these models were re-evaluated using a grid-search function with a maximum of 30 iterations from 100 random vectors of the initial estimation values of a 1-class model (Supplementary Table 3). All the models from the class-specific variance-covariance structure had very low BIC and AIC values, even though models with an increasing number of classes had the lowest BIC and AIC values. To select the appropriate model with the optimal number of classes, we assessed the discrimination power of the models using their entropy values and also assessed the relevance of the identified trajectories using their assigned posterior probability.

A model with an entropy value greater than 0.70, an average posterior probability of class membership of ≥ 70%, and at least n=10 of the population assigned to the minor class was selected as the final model with the appropriate number of latent classes. Based on the AIC, BIC, and entropy values in Supplementary Table 3, the model with 4 latent ketosis trajectory classes under grid search was deemed to be the best fit (i.e., lower BIC, AIC, and SABIC values) when compared to models with 3 or fewer and 5 or more latent classes (Supplementary Tables 2 and 3). The entropy of the model was 0.74, indicating good separation between the classes.

The four latent ketosis classes identified were:

1. Unsustained Nutritional Ketosis (UNK): This class included 27 individuals. These individuals experienced brief periods of ketosis but did not maintain it consistently over time.
2. Low Nutritional Ketosis (LNK): This class included 105 individuals who had low but stable levels of ketones throughout the study period.
3. Moderate Nutritional Ketosis (MNK): This class comprised 99 individuals who started with moderate levels of ketones that decreased over time.
4. Sustained Nutritional Ketosis (SNK): This class included 17 individuals who maintained high levels of ketones consistently over the study period.

The selection of these four classes was based on their ability to capture distinct patterns of ketosis over time, providing insights into the variability of ketone levels among different individuals. Supplementary Table 4 includes a completed GRoLTS (Guidelines for Reporting on Latent Trajectory Studies) checklist for the latent class trajectory analysis, ensuring transparency and reproducibility of the results.

|  | G | loglink | conv | npm | AIC | BIC | SABIC | entropy | ICL |
| --- | --- | --- | --- | --- | --- | --- | --- | --- | --- |
| pct_ketone_linear_2yr | 1 | 1.00E+09 | 4 | 6 | 2.00E+09 | 2.00E+09 | 2.00E+09 | 1 | 2.00E+09 |
| pct_ketone_quad_2yr | 1 | 2.78E+01 | 1 | 10 | -3.56E+01 | 5.63E-02 | -3.16E+01 | 1 | 5.63E-02 |
| pct_ketone_beta_2yr | 1 | 1.43E+02 | 2 | 8 | -2.70E+02 | -2.42E+02 | -2.67E+02 | 1 | -2.42E+02 |
| pct_ketone_spl_2yr | 1 | 5.85E+02 | 1 | 11 | -1.15E+03 | -1.11E+03 | -1.14E+03 | 1 | -1.11E+03 |
| pct_ketone_spl5q_2yr | 1 | 7.27E+02 | 1 | 11 | -1.43E+03 | -1.39E+03 | -1.43E+03 | 1 | -1.39E+03 |
| pct_ketone_spl7q_2yr | 1 | 1.00E+03 | 1 | 13 | -1.97E+03 | -1.93E+03 | -1.97E+03 | 1 | -1.93E+03 |
| pct_ketone_spl5e_2yr | 1 | 5.85E+02 | 1 | 11 | -1.15E+03 | -1.11E+03 | -1.14E+03 | 1 | -1.11E+03 |
| pct_ketone_spl7e_2yr | 1 | 6.82E+02 | 1 | 13 | -1.34E+03 | -1.29E+03 | -1.33E+03 | 1 | -1.29E+03 |
| pct_ketone_spl7q_1_2yr_2_cov | 1 | 952.7223 | 1 | 20 | -1865.445 | -1795.749 | -1859.144 | 1 | -1795.749 |

**Supplementary Table 1**. Model specification and parameters for one-class models using different link functions

**Note:** loglink= logarithmic link function, conv = model convergence, npm = number of parameters, AIC = Akaike Information Criterion; BIC = Bayesian Information Criterion; SABIC = Sample-size adjusted Bayesian Information Criterion, ICL=Integrated Completed Likelihood. Model selection was guided by fit indices (AIC, BIC, SABIC). The optimal model was chosen based on the lowest AIC and BIC values.

Missing data were handled using full information maximum likelihood (FIML).

**Supplementary Table 2.** Model specification and parameters for models up to 6 classes using quadratic 1-splines function with 7 nodes and a common variance-covariance structure

|  | G | loglink | AIC | BIC | SABIC | entropy | ICL | %class 1 | %class 2 | %class 3 | %class 4 | %class 5 | %class 6 |
| --- | --- | --- | --- | --- | --- | --- | --- | --- | --- | --- | --- | --- | --- |
| pct_ketone_spl7q_n_2_2yr_2 | 2 | 1012.35 | -1992.7 | -1935.7 | -1986.4 | 0.699 | -2411.6 | 57.85 | 42.15 |  |  |  |  |
| pct_ketone_spl7q_n_3_2yr_2 | 3 | 1014.34 | -1990.7 | -1922.9 | -1983.2 | 0.634 | -2354.3 | 27.979 | 46.36 | 25.67 |  |  |  |
| pct_ketone_spl7q_n_4_2yr_2 | 4 | 1017.17 | -1990.3 | -1911.9 | -1981.7 | 0.616 | -2321.4 | 19.16 | 39.46 | 34.87 | 6.51 |  |  |
| pct_ketone_spl7q_n_5_2yr_2 | 5 | 1019.70 | -1989.4 | -1900.3 | -1979.5 | 0.627 | -2295.5 | 7.28 | 23.75 | 27.97 | 29.50 | 11.49 |  |
| pct_ketone_spl7q_n_6_2yr_2 | 6 | 1022.67 | -1989.3 | -1889.5 | -1978.3 | 0.657 | -2283.9 | 8.43 | 19.54 | 29.12 | 18.01 | 22.22 | 2.68 |

**Note:** loglink= logarithmic link function, conv = model convergence, npm = number of parameters, AIC = Akaike Information Criterion; BIC = Bayesian Information Criterion; SABIC = Sample-size adjusted Bayesian Information Criterion, ICL=Integrated Completed Likelihood. Model selection was guided by fit indices (AIC, BIC, SABIC), entropy (measure of class separation), and theoretical interpretability. The optimal model was chosen based on the lowest AIC and BIC values and clinical relevance.

Entropy values range from 0 to 1, with higher values indicating better classification accuracy. Class membership probabilities represent the likelihood of individuals being assigned to a specific trajectory group. Missing data were handled using full information maximum likelihood (FIML).

**Supplementary Table 3.** Model specification and parameters for models up to 6 classes using quadratic 1-splines function with 7 nodes and a

class-specific variance-covariance structure and grid-search

|  | G | loglink | AIC | BIC | SABIC | entropy | ICL | %class 1 | %class 2 | %class 3 | %class 4 | %class 5 | %class 6 |
| --- | --- | --- | --- | --- | --- | --- | --- | --- | --- | --- | --- | --- | --- |
| pct_ketone_spl7q_n_2_2yr | 2 | 1012.4 | -1992.7 | -1930.2 | -1984.1 | 0.703 | -2406.8 | 58.62 | 41.38 |  |  |  |  |
| pct_ketone_spl7q_n_2_T_grid_2_yr | 2 | 1012.4 | -1990.8 | -1930.2 | -1984.1 | 0.703 | -2406.8 | 58.62 | 41.37 |  |  |  |  |
| pct_ketone_spl7q_n_3_2yr | 3 | 1014.8 | -1987.5 | -1912.7 | -1979.2 | 0.640 | -2339.6 | 25.67 | 13.03 | 61.30 |  |  |  |
| pct_ketone_spl7q_n_3_T_grid_2_yr | 3 | 1016.5 | -1991.0 | -1916.1 | -1982.7 | 0.767 | -2385.1 | 6.130 | 42.15 | 51.72 |  |  |  |
| pct_ketone_spl7q_n_4_2yr | 4 | 1017.3 | -1984.5 | -1895.4 | -1974.7 | 0.642 | -2314.6 | 28.74 | 16.86 | 45.98 | 8.43 |  |  |
| pct_ketone_spl7q_n_4_T_grid_2_yr | 4 | 1021.8 | -1993.6 | -1904.5 | -1983.8 | 0.740 | -2346.3 | 42.91 | 39.46 | 11.11 | 6.51 |  |  |
| pct_ketone_spl7q_n_5_T_2_yr | 5 | 1022.9 | -1987.8 | -1884.4 | -1976.3 | 0.694 | -2301.8 | 32.57 | 12.26 | 4.98 | 9.96 | 40.23 |  |
| pct_ketone_spl7q_n_5_T_grid_2_yr | 5 | 1022.9 | -1987.8 | -1884.4 | -1976.3 | 0.694 | -2301.7 | 4.98 | 9.96 | 40.23 | 12.26 | 32.57 |  |
| pct_ketone_spl7q_n_6_T_2_yr | 6 | 1027.4 | -1988.9 | -1871.2 | -1975.9 | 0.694 | -2273.5 | 8.05 | 10.73 | 24.52 | 27.59 | 14.56 | 14.56 |
| pct_ketone_spl7q_n_6_T_grid_2_yr | 6 | 1025.9 | -1985.9 | -1868.2 | -1972.8 | 0.717 | -2283.1 | 37.55 | 12.64 | 6.51 | 0.38 | 31.80 | 11.11 |

**Note:** loglink= logarithmic link function, conv = model convergence, npm = number of parameters, AIC = Akaike Information Criterion; BIC = Bayesian Information Criterion; SABIC = Sample-size adjusted Bayesian Information Criterion, ICL=Integrated Completed Likelihood. Model selection was guided by fit indices (AIC, BIC, SABIC), entropy (measure of class separation), and theoretical interpretability. The optimal model was chosen based on the lowest AIC and BIC values and clinical relevance.

Entropy values range from 0 to 1, with higher values indicating better classification accuracy. Class membership probabilities represent the likelihood of individuals being assigned to a specific trajectory group. Missing data were handled using full information maximum likelihood (FIML).

**Supplementary Table 4.** Guidelines for Reporting on Latent Trajectory Studies (GRoLTS) Checklist

| **No.** | **Checklist Items** | **Yes/No** |
| --- | --- | --- |
| **1.** | **Is the metric of time used in the statistical model reported?**  Time was treated as time-structured data | Yes |
| **2.** | **Is information presented about the mean and variance of time within a wave?**  Time was treated as time-structured data | No |
| **3a.** | **Is the missing data mechanism reported?**  Details on missing data and how it was handled are included in the method section | Yes |
| **3b.** | **Is a description provided of what variables are related to attrition/missing data?** | Yes |
| **3c.** | **Is a description provided of how missing data in the analyses were dealt with?** | Yes |
| **4.** | **Is information about the distribution of the observed variables included?** | Yes |
| **5.** | **Is the software mentioned?** | Yes |
| **6a.** | **Are alternative specifications of within-class heterogeneity considered (e.g., LGCA vs. LGMM) and clearly documented? If not, was sufficient justification provided as to eliminate certain specifications from consideration?** | Yes |
| **6b.** | **Are alternative specifications of the between-class differences in variance-covariance matrix structure considered and clearly documented? If not, was sufficient justification provided as to eliminate certain specifications from consideration?** | Yes |
| **7.** | **Are alternative shape/functional forms of the trajectories described?** | Yes |
| **8.** | **If covariates have been used, can analyses still be replicated?** | Yes |
| **9.** | **Is information reported about the number of random start values and final iterations included?** | Yes |
| **10.** | **Are the model comparison (and selection) tools described from a statistical perspective?** | Yes |
| **11.** | **Are the total number of fitted models reported, including a one-class solution?** | Yes |
| **12.** | **Are the number of cases per class reported for each model (absolute sample size, or proportion)?** | Yes |
| **13.** | **If classification of cases in a trajectory is the goal, is entropy reported?** | Yes |
| **14a.** | **Is a plot included with the estimated mean trajectories of the final solution?** | Yes |
| **14b.** | **Are plots included with the estimated mean trajectories for each model?** | No |
| **14c.** | **Is a plot included of the combination of estimated means of the final model and the observed individual trajectories split out for each latent class?** | No |
| **15.** | **Are characteristics of the final class solution numerically described (i.e., means, SD/SE, n, CI, etc)?** | Yes |
| **16.** | **Are the syntax files available (either in the appendix, supplementary materials, or from the authors)?** Upon request from the authors | Yes |
